# The positive impacts of external focus of attention on performance, fatigue, and affect on a fatiguing task

**DOI:** 10.3389/fpsyg.2026.1753471

**Published:** 2026-03-19

**Authors:** Marcos Daou, Emily Gannon, Melody Winepol, Alexis Coville, Blake Simpson, Timothy Rotarius, Justin Guilkey, Greg Martel

**Affiliations:** Department of Kinesiology, Coastal Carolina University, Conway, SC, United States

**Keywords:** fatigue, focus of attention, isometric task, motor performance, pleasantness

## Abstract

Creating strategies to improve performance and delay fatigue is an important goal in the field of coaching and sports performance. These improvements are typically reached through training, nutritional, and psychological strategies. The present study aimed to investigate how attentional focus affects motor performance, physiological fatigue, muscle activation, muscle oxygenation, perceived exertion, and affect (pleasantness/unpleasantness) on fatiguing wall-sit tasks. Sixty physically active participants completed two wall-sit tasks until failure in a counterbalanced order, while adopting either an internal or external FoA. Our goal was to examine how FoA affected time to failure, physiological fatigue (MDF), muscle oxygenation (SmO_2_), perceived exertion (RPE), and pleasantness during the two tasks. Results revealed that subjects prolonged time to failure when adopting an external FoA. Moreover, the external focus reduced RPE and unpleasantness during the wall-sit task relative to the internal FoA. However, no significant differences were observed between FoA conditions for MDF, muscle activation, or SmO2 despite significant muscle alterations in both conditions from the beginning to the end of each wall-sit task. In conclusion, adopting an external FoA improved time to failure in the wall-sit task and this result may be associated with reduced RPE and decreased feelings of unpleasantness close to the end of the task.

## 1 Introduction

On a classical concept of skill performance, Guthrie (1952) mentioned that it “consists in the ability to bring about some end result with maximum certainty of goal achievement and minimum outlay of energy” (p. 136). Wulf et al. (2010) added to it by mentioning that movements are performed fluently and efficiently with relatively little physical or mental effort. For instance, an expert volleyball player can generate more speed and force while maintaining accuracy and precision levels as compared to a novice, even though the expert utilizes less muscle activation. Expert runners can run faster and cover more miles while wasting less energy than a novice. Thus, with practice, movements are produced and organized with less muscular energy or physical effort (e.g., Lay et al., 2002). Therefore, creating practical ways to improve motor performance in a way that the skill can be performed efficiently (more accuracy with less effort) is one of the goals that coaches, athletes, and exercisers constantly seek to obtain. Questions related to what information is relevant and irrelevant to attend to while performing motor skills become crucial for superior performance since it involves psychological processes that may require the brain to use more or less energy while completing a motor task. Knowing how and where to focus one's attention while learning and performing motor tasks is key to improving performance while at the same time distracting themselves from the detrimental sensations of fatigue and unpleasantness during the task.

Focus of attention (FoA) and performance is a theory that demonstrates how performers can use their attention to reach maximum performance while executing the skill in an efficient way. Adopting different FoA may impact individuals' behaviors differently while performing skills, movements, and motor tasks. While performing motor tasks, performers can either adopt an internal or external FoA (Wulf, 2007a). An internal FoA is manifested when performers direct their attention to their own bodies while performing a motor task (e.g., thinking about the position of the feet, rotation of the arm, or the force being utilized to execute an action). On the other hand, an external FoA is adopted when a performer focuses their attention externally from their bodies but related to the goal of the task or trajectory of the movement. For example, looking at the rim while performing a jump shot in basketball, or visualizing the trajectory of a throw while the ball travels toward the catcher's glove while pitching a baseball (Wulf, 2007a). Over the past 20 years, research in the field of exercise and sports science has increasingly demonstrated the importance of FoA and human performance. Previous research has consistently shown the benefit of external FoA for superior skill performance and learning. For example, Chua et al. (2021) showed through a meta-analytic study that an external focus of attention was superior to an internal focus whether considering tests of motor performance or learning, and regardless of age, health condition, and level of skill expertise. Following the authors outcome, if individuals are willing to improve performance and learning, external focus would be the best approach (Chua et al., 2021). Additionally, studies have shown the benefit of an external FoA on accuracy for hitting targets and reaching a movement goal (Bell and Hardy, 2009), reaching and producing a specific range of force (Lohse et al., 2011), producing movement speed (Porter et al., 2010), and improving different types of motor skills (Wulf, 2007a; Lohse and Sherwood, 2012). Moreover, previous research has demonstrated that when individuals receive instructions that encourage them to focus externally, their performance tends to improve compared to situations where no attentional guidance was given (McNevin and Wulf, 2002; Wulf and McNevin, 2003; Wulf et al., 2003). Additionally, Aiken and Becker (2022) found that external focus of attention always generated superior performance on a simple discrete task, however, internal FoA was detrimental to the task execution only during test phase, therefore, both internal and external FoA facilitated learning during the acquisition phase. Lastly, Strick et al. (2023) found that external focus positively impacted standing long jumps regardless of the type of internal focus it was adopted (thinking about the toes, knees, hips, arms; and control).

The benefits of an external FoA rely on how individuals utilize their cortical resources in the working memory while performing and learning motor skills. Working memory was found to be a central part of human cognition while performing cognitive and various demanding tasks. It is understood as a “bridge” between brain processes and situational demands. Ericsson and Delaney (1999) affirmed that they have not found a human activity that did not impact working memory. When an external FoA is utilized, a more automatic process is adopted, and less cortical resources are utilized in the working memory. On the other hand, when adopting an internal FoA, motor tasks become more conscious, and it increases the cortical resources demand that will eventually slow down the processes, decreasing performance. This explanation is in line with the constrained action hypothesis (McNevin et al., 2003). According to this theory, an internal FoA interferes with automatic control by forcing the performer to consciously monitor the movement. This disrupts the natural flow of the motor system. In contrast, an external FoA helps the motor system to self-organize more efficiently, allowing for quicker and more natural movement execution (Wulf et al., 2001). Interestingly, Qin et al. (2025) and Kuhn et al. (2021) investigated how focus of attention impacted performance and brain efficiency to complete an arrow shooting task and a pedal-tracking task respectively. The authors did not find any benefit of focus of attention on performance; however, they saw that the brain worked more efficiently when external focus was adopted.

Despite a robust number of studies showing the benefit of external FoA, not all studies have demonstrated this effect. There is not a “one-size-fits-all” perspective related to FoA and performance. Some research has suggested that an internal FoA may benefit beginners under certain conditions (Beilock et al., 2004; Castaneda and Gray, 2007). This follows the rationale that when performers are learning a new motor task or skill, the first step is to create a theory, rule, or norm of the task that is being learned to progress to a procedural knowledge. After a thorough creation and representation of the skill in the motor cortex, then individuals are “free” to apply this motor task theory into an applicable reality. In addition, novice athletes often default to an internal FoA, especially when learning new or complex tasks. They tend to focus their attention to their own bodies in an attempt to assimilate and coordinate the movements (Beilock and Carr, 2001; Beilock et al., 2004). This is a part of a cognitive strategy called “reinvestment” (Masters and Maxwell, 2008), where in order to automatize a skill, humans have a tendency to create a theory of the skill first. On the other hand, an external FoA is understood as a great “technique” to facilitate motor performance and learning, while speeding up learning processes with greater movement economy (Wulf, 2007a,b). According to the constrained action hypothesis (Wulf, 2007a,b), an internal FoA creates top-down control that interferes with smooth automatic movement. Supporting research has shown that an external FoA is linked to reduced muscle stiffness (Lohse et al., 2011) and more adaptive movement patterns (Lohse et al., 2011).

Previous studies in the field of motor learning and control revealed the importance of examining FoA and its effects on force production and performance (Lohse and Sherwood, 2012; Marchant, 2011; Marchant et al., 2009a,b; Lohse et al., 2010; Wulf et al., 2010). Findings indicated that when individuals focused externally to their bodies while performing skills and movements (e.g., onto movement outcomes), it was shown to be more beneficial than internally focused instructions (focusing on the body parts while movements were executed). The benefits of an external FoA were observed on tasks and skills that involved maximal and accurate force production, in addition to force maintenance in continued tasks. Specifically, electromyography (EMG) has been utilized to examine muscle activation, efficiency, and physiological fatigue (MDF). As an example, Wulf et al. (2001) showed signs of automaticity linked to a reduced muscular activity with an external FoA. This characteristic seemed to be linked to advanced levels of performance as represented by a superior movement and skill outcomes (more accurate, faster time, and more weight lifted, for example). Vance et al. (2004) showed that a reduced EMG response of the biceps brachii was generated when participants adopted an external FoA while lifting more weight. In the same way, Marchant et al. (2008) found that the movement was executed more efficiently by generating more force, while presenting less muscle activation under an external FoA. Potential mechanisms were identified as the reasons why an external FoA may have promoted superior performance and movement efficiency. Lohse et al. (2010) assessed neuromuscular correlates during a target-force production task. Internally focused instructions resulted in greater error in producing the target force as well as leading to reduced muscular efficiency compared to externally focused instructions. Wulf et al. (2010) found that an external FoA was correlated with more effective maintenance of submaximal force production, whereas an internal FoA negatively impacted muscular endurance through inefficient movement. The effects of differing FoA on tasks requiring isometric force production have also been investigated in previous studies that focused on changes in intermuscular and intramuscular coordination (Lohse et al., 2011). The authors showed that an internal FoA led to increased errors in isometric force production compared to an external FoA. Therefore, the study by Lohse et al. (2011), and Lohse (2012) found that adopting an internal FoA led to less effective movement (i.e., increased absolute error) and to less efficient intermuscular and intramuscular coordination during tasks that require isometric force production.

In addition, studies have investigated the effects of FoA on skill performance, with multiple studies finding benefits when participants adopted an external FoA. For example, Zachry et al. (2005) observed that basketball players improved their free-throw accuracy and exhibited lower muscle activation when focusing on the rim rather than their arm movement. Similar effects were reported in elbow flexion exercises (Vance et al., 2004; Marchant et al., 2009a,b) and vertical jump tasks (Wulf et al., 2010; Porter et al., 2010). Additionally, Lohse et al. (2010) showed that an external FoA made individuals improve their accuracy while decreasing EMG activity in the triceps of the throwing arm in a dart-throwing experiment. Lastly, gravitating toward the strength and conditioning realm, Marchant et al. (2009a,b) had participants performing biceps curls while receiving either internal or external FoA instructions. The internal cue asked them to focus on contracting their biceps, while the external cue directed attention to the movement of the bar. Results revealed that the external FoA condition led to higher force production, even though biceps muscle activation (measured by EMG) was lower than in the internal FoA condition. These findings suggest that focusing externally allows for more efficient recruitment of motor units, possibly reducing unnecessary muscle tension. Attentional strategies might therefore be useful not only for improving performance but also for making training more efficient. Positive results were seen for the pectoralis major and triceps muscles during bench press exercises (Snyder and Fry, 2012; Calatayud et al., 2016), particularly at moderate intensities. Moreover, research involving maximal performance tasks, such as vertical jumping, has revealed that the displacement of the center of mass was greater and muscle activity in the lower limbs was reduced when adopting an external FoA (Wulf et al., 2010). Lastly, an external FoA appears to positively impact acute physiological responses during running, such as heart rate (Connolly and Janelle, 2003) and metabolic efficiency (Schücker et al., 2009). Lastly, Porter et al. (2010) showed that external focus instructions reduced the time taken to complete a whole-body agility task, while Chen et al. (2023) investigated the effects of foci of attention in a geriatric population showed that the overall effect of an external focus resulted in better motor performance, especially in postural control than an internal focus among older adults.

Recently, a new line of research has been emerging utilizing the same FoA approach in the field of strength and conditioning. Beyond enhancing force, an external FoA has also been associated with better muscular endurance. For instance, Marchant et al. (2011) had subjects performing bench press repetitions to failure. Results showed that individuals adopting external FoA instructions completed more repetitions than those who received internal or neutral cues. In addition, Nolan (2011) suggests that an external FoA may be more beneficial for isometric muscle endurance than an internal FoA. Their findings revealed that subjects were able to perform wall-sit tasks for a significantly longer duration in trials that utilized external FoA instructions. This approach may also be relevant to strength and conditioning, such as improving resistance training, speed development, jumping, and balance control. For example, Schücker et al. (2009) reported improved running efficiency under external FoA conditions. From a practical point of view, coaches should pay close attention to the verbal instructions they give to athletes. Avoiding cues that highlight body parts or internal movements may help prevent athletes from focusing internally, which could limit performance (Makaruk and Porter, 2014). Contrary to the finds of Makaruk and Porter (2014), Schoenfeld et al. (2018) examined the effects of FoA on resistance training across 8 weeks in untrained individuals. Results showed that those who used an internal FoA (concentrating on contracting their biceps muscles) achieved greater hypertrophy in the elbow flexors compared to the external focus group. However, this difference was not seen in the quadriceps muscle group. Schoenfeld and Contreras (2016) also showed the benefit of an internal FoA on muscle activation and hypertrophy, particularly when lifting moderate loads (~50% of 1-Repetition Maximum; 1-RM). This study is in line with the “mind-muscle connection” perspective that is popular among bodybuilders and may help stimulate muscle growth when training at lower intensities.

Despite these consistent findings in power-based tasks, relatively few studies have explored the effects of FoA on exercises requiring muscle endurance. Marchant et al. (2011) examined the effects of FoA on muscular endurance in trained individuals performing exercise routines. They measured repetitions to failure for various weight training exercises such as bench press and free squat. Results revealed that an external FoA led to completion of a higher number of repetitions and that an external FoA had even more impact as movement complexity increased. For simple exercises, or early in practice, an internal FoA showed positive effects as well. Lohse and Sherwood (2011) demonstrated that an external FoA improved performance and reduced RPE, but the study did not examine neuromuscular recruitment mechanisms. To extend the work of Lohse and Sherwood (2011), the current study added physiological measurements to investigate mechanisms behind how differing FoA may affect performance on isometric wall-sit tasks. The current study used surface EMG to assess muscle activation and physiological fatigue (MDF), muscle oxygenation (SmO2) via near-infrared spectroscopy (NIRS), unpleasantness (via the Feelings Scale), and the Borg RPE scale to assess perceived fatigue (Borg, 1998). These measures were assessed at the beginning and end of each wall-sit task. Based on the background provided, the aim of this experiment was to examine how FoA (internal vs. external) affected time to failure, muscle activation, MDF, SmO2, RPE, and unpleasantness while individuals performed two fatiguing, isometric wall-sit tasks. We hypothesized that (H1) an external FoA would prolong time to failure during the wall-sit task (perform better) relative to an internal FoA. In addition, we hypothesized that (H2) an external FoA would be related to decreased MDF, decreased SmO2, lower RPE, and less unpleasantness toward the wall-sit task as compared to an internal FoA.

## 2 Material and methods

### 2.1 Participants

Sixty young (34 females, 26 males), physically active with ages between 18 and 30 years (Mean age: 21.03 ± 1.37 years), volunteered to participate in this study after consenting to a protocol approved by the Coastal Carolina University Institutional Review Board (protocol #2024.175) (see Table 1). Participants were recruited from university courses and by word of mouth and were compensated with course credit. All subjects reported engaging in strength training exercises at least 2 days per week and affirmed not having any physical or psychological limitations to participate in wall-sit tasks. Sample size was determined with an a priori power calculation (G^*^Power software—Version 3.1; Faul et al., 2009) providing 80% statistical power (α = 0.05) to detect a moderate-sized within-subject effect size (Cohen's dz = 0.50) for time to failure. Additionally, a moderate-sized (partial η^2^ = 0.09) Within-subjects x Between-subject interaction for physiological fatigue and blood oxygenation was performed providing a sample size of 44 participants.

**Table 1:** Demographics.

**Number of participants**	**Gender**	**Age**	**Number of times participants exercised per week**
Sixty (60)	34 females 26 males	21.03 years old (1.37 std. dev)	2–3 times 12 participants 4–5 times 34 students 6 or more times 14 students

### 2.2 Design

**Figure 1:**
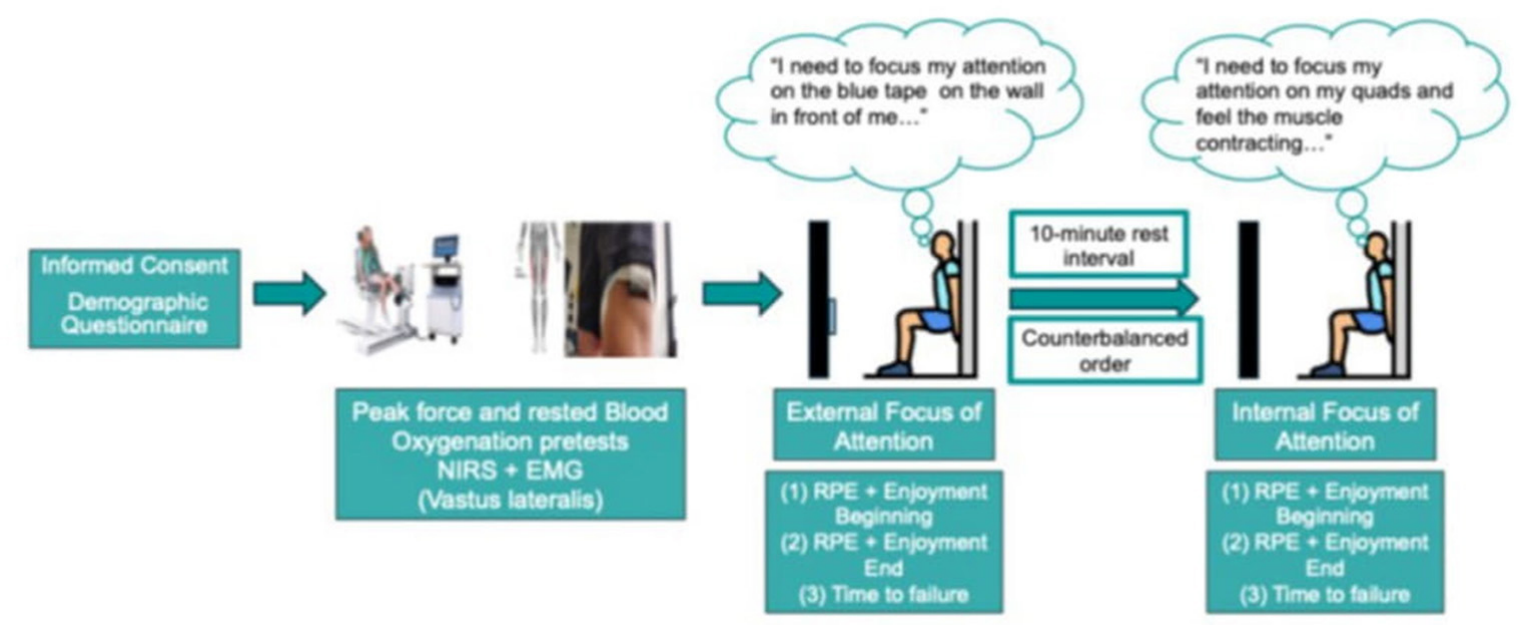
Experiment design. From left to right, the figure represents the order of events of the research design.

Participants were required to come to the exercise physiology lab for one visit that took approximately 60 min. Once they arrived, we had them signed the informed consent, filling out demographic questionnaires with personal information about fitness level, injury history, sports participation, etc., then were told about the nature of the wall-sit tasks and procedures prior to beginning. Importantly, only individuals that were physically active and had no physical or psychological restrictions were able to participate (all individuals were asked not to exercise for at least 12 h before the experiment). Since one of the goals of this study was to replicate the experiment conducted by Lohse and Sherwood (2011), we maintained the same design, however with the addition of physiological measurements to analyze muscle activation and MDF as well as SmO_2_ during the tasks. Because of the nature of the physiological measurements during the wall-sit tasks, maximal isometric force production (peak force) was determined prior to the wall-sit tasks using a Biodex dynamometer; resting SmO_2_ was also obtained before the wall-sit tasks (Figure 1).

All participants were required to perform two wall-sit tasks until failure while adopting either an internal or external FoA in a counterbalanced order. A 10-min rest interval was provided between the two tasks to reduce fatigue. Besides time to failure, four other variables were collected while subjects perform both tasks: physiological fatigue (MDF), muscle oxygenation (SmO_2_), perceived exertion (RPE), and affect (feeling scale). Importantly, to create points of references to measure the previous variables, we defined to epochs (times) to be analyzed. (1) beginning of the task was treated as the first 20 s of the wall-sit, while the end of task was treated within the last 20 s of wall-sit task. The subjects reported being familiar with the task from previous athletic experience (even though they had not performed the task within the previous 5 years). Nevertheless, all subjects completed a short habituation trial (lasting10 s) prior to the start of the experimental trials.

After completing the habituation trial, all subjects received the following instructions, “Today you will perform two wall-sit tasks until failure under two different conditions. We will monitor the physiological responses from your quadriceps (vastus lateralis) during each task. You will only do two trials, and you will have a 10-min resting period in between each one of them. The reason for a limited number of trials and a long rest period is because we want you to try your best to hold the wall-sit posture for as long as possible. Do not try to pace yourself, and try your best on each trial. It is completely up to you when you want to end a trial. When you feel like you cannot hold the posture any longer, just say “Done” and then stand up or sit all the way down and the trial will be over. In addition, if you fail to keep the knees and hips angles within the 90° ± 5° range your attempt will be terminated and it will be understood as the time to failure However, we do want you to make the trial last as long as possible. Following these instructions, subjects began the experimental trials. Both FoA conditions were completed by all participants in the same session, but in a randomized, counterbalanced order.

### 2.3 Wall-sit tasks and FoA instructions

The following instructions and conditions were replicated from Lohse et al. (2011):

**Internal FoA Instructions:** For the internal focus, subjects were reminded to visually focus on the small fixation dot (2 cm) on the rolling wall in front of them at the beginning of the trial as a cue to maintain the back flat against the wall, but as the trial started “to mentally focus on the position of your thighs, trying to keep them parallel to the floor to minimize any movement up and down.” Once the trial began, subjects were given feedback on the time every 30 s and reminded of their focus by being told to “focus on the position of your thighs, trying to keep them parallel to the floor.

**External FoA Instructions:** For the external focus, subjects were reminded to visually focus on the small fixation dot (2 cm) on the rolling wall in front of them at the beginning of the trial as a cue to maintain the back flat against the wall, but as the trial started, “to visually focus on the “knee-height” blue tape (4 cm x 8 cm) placed on the rolling wall in front of them and to mentally focus on an imaginary line between the knee and the blue tape in front of you, trying to keep the lines parallel to the floor to minimize any movement up and down.” Once the trial began, subjects were given feedback on the time every 30 s and “to focus on the blue tape in front of them and on the position of the lines, trying to visualize them parallel to the floor.”

### 2.4 Wall-sit task procedures

For all trials, after the instructions were given, subjects were asked for verbal confirmation that they understood the instructions and were required to paraphrase the instructions back to the experimenters. After verifying that subjects understood the instructions, they began their first experimental trial. All participants were instructed to focus on a small fixation dot (2 cm) on the rolling wall in front of them (ten feet away) before the trial started (regardless of the condition) to make sure they would “learn” and keep their back flat against the wall during the duration of the tasks and not leaning or “looking” somewhere else. Then, different instructions were provided to each focus of attention condition. Importantly, for the external focus of attention, a blue tape (4 cm x 8 cm) cue was placed on the rolling wall in front of the participants and removed for the Internal FoA condition.

Subjects then walked their feet out away from the wall until their knees and ankles were in the 90° ± 5° range and the experimenter started the trial by starting the hand timer (S141, Seiko, Tokyo, Japan) and saying, “Begin”. The experimenter reminded subjects of the experimental FoA every 30 s. The experimenter visually monitored the position of the subjects' legs to make sure they were within the 90° ± 5° tolerance range as well as the subjects' gaze, making sure they were staying visually focused on the fixation point throughout the trial. Subjects verbally stopped the trial by saying “Done” when they reached the point of voluntary exhaustion. The hand timer was stopped exactly at this time. The amount of time from the start of the trial to the end of the trial was defined as “time to failure” (measured in seconds).

### 2.5 Time to failure (performance)

Subjects first had reflective anatomical markers placed on the knee (at the lateral condyle of the femur) and the hip (greater trochanter of the femur) on each leg to create a point of reference to keep constant verification whether the subjects were keeping the correct wall-sit technique position. Analog goniometers (Prestige Medical, Northridge, CA, USA) were used to make sure the knee and ankle angles were within 90° ± 5° at the start of the trial. After verifying that subjects understood the instructions, they took position to start their first experimental trial. Subjects stood with their back flat against the wall and visually focused on the small fixation dot (2 cm) on the rolling wall in front of them as a cue to keep their back flat against the wall before the trial start. Subjects then walked their feet out away from the wall until their knees and ankles were in the 90° ± 5° range and the experimenter started the trial by hitting the hand timer (S141, Seiko, Tokyo, Japan) and saying “Begin.” In every 30 s, the experimenter would remind subjects of the experimental focus and update subjects on the time. The experimenter visually monitored the position of the subjects' legs, within the 90° ± 5° tolerance, and subjects gaze throughout the trial. Participants would verbally stop the trial by saying, “Done” when they reached the point of voluntary exhaustion. Additionally, fail to keep the knees and hip angles within the 90° ± 5° correct technique range automatically terminated the attempt and was defined as *time to failure*. All and all, the time from the start of the trial to the end of the trial (or until the participants could not sustain the 90° ± 5° correct technique position) was defined as *time to failure* (measured in seconds by the Seiko141 stopwatch).

### 2.6 RPE and measures of unpleasantness

Perceived fatigue (RPE) and unpleasantness were verbally assessed by the experimenter by asking the participants about their RPE using a 15-point Borg Scale (Borg, 1998) and the Feelings Scale (unpleasantness), respectively. Subjects provided subjective RPE and unpleasantness scores for how difficult and how unpleasant participants perceived the wall-sit tasks in the first 20 s of the trial and at the very end of the trial. Importantly, a laminated printed version of each scale (Borg and Feelings Scale) was shown to the participants, and they were required to tell a number that best represented their RPE and unpleasantness at the beginning and at the end of the wall-sit tasks (e.g., “It feels like a 15 right now” or “it feels like a −2”). Between the counterbalanced trials, subjects were allowed to rest for 10 min or four times the length of the previous trial (whichever time resulted in a longer rest period). Subjects were told that this resting time was the minimum time they could rest between trials, but they could take more time if they did not feel fully recovered.

### 2.7 Muscle activation and MDF measurements

Surface EMG was measured continuously throughout each wall-sit task. A double differential EMG electrode (Trigno Avanti, Delsys, Natick, MA) was placed on the vastus lateralis of the dominant leg approximately 3–5 cm superior to the patella at an oblique angle just lateral to the midline of the belly of the muscle, parallel to the direction of the muscle fibers (Cram et al., 1998). Inter-electrode resistance was reduced by shaving and cleaning the surface of the skin with alcohol prior to placing the electrode over the muscle. The EMG signal was checked for motion artifact or noise by moving the leg and tapping the skin near the electrode. The electrode was re-applied if any motion artifact was observed.

Maximum voluntary isometric contractions (MVICs) were assessed to determine peak force before the wall-sit tasks using an isokinetic dynamometer (Biodex System 3, Medical Systems, Shirley, NY). The isokinetic dynamometer was adjusted for each participant according to the manufacturer's recommendations to ensure similar body positions of all subjects. Before beginning the wall-sit tasks, participants were asked to complete three (3) MVICs for the knee extensors with their knee fixed at 90 degrees. Participants were instructed to push against the dynamometer attachment and attempt to extend their knee with as much force as possible for 5 s. Each MVIC was followed by 2 min of recovery until 3 MVICs were completed. The peak force score was averaged utilizing the middle 2 s of each trial.

The raw EMG signal was collected at a sampling frequency of 2,000 Hz, digitally filtered using a band-pass filter (10–500 Hz), and smoothed using a 50-millisecond root-mean-squared (RMS) window (Fukuda et al., 2010). For each trial, the RMS values were sampled at 1 s intervals, time aligned with the onset of each condition and the first 20 s and the last 20 s of each wall-sit were averaged. The RMS signal for the first 20 s and last 20 s for each participant were then expressed as a percentage of the MVIC. For each condition, power spectral analysis was performed (Fast-Fourier transformation was performed using 1,024 data points), and the median power frequency (MDF) was calculated (Phinyomark et al., 2012). The first 20 s and last 20 s of each condition were averaged for each condition and the difference between the beginning and end of the wall-sit condition was used to assess muscular fatigue (MDF). Two-way analyses of variance (ANOVA) with two repeated measures (Condition, Time) were conducted to determine differences in EMG between each condition. A Student-Newman-Keul's *post-hoc* test was used, if needed.

### 2.8 SmO_2_

SmO_2_ was collected using a single-channel continuous-wave near-infrared spectroscopy (NIRS) monitor at a frequency of 0.5 Hz. The NIRS device was covered with a shield to limit external light sources and affixed midway between the anterior iliac spine and the superior border of the patella over the belly of the vastus lateralis on the non-dominant leg. Prior to the wall sit, SmO_2_ was collected during a 3-min seated rest and was averaged over the final 30 s to determine a resting, baseline SmO_2_. Additionally, SmO_2_ was collected over the entire duration of the wall-sit tasks and was averaged over the first 15 s and the final 15 s of the wall-sit task. Because continuous-wave NIRS monitors cannot determine absolute concentrations, reported SmO_2_ data are expressed as a change from the baseline data (Δ BSL) in arbitrary units (AU). To understand the overall hypoxic stress of each FoA condition and to account for differences in wall sit times to failure, the SmO_2_ area under the curve (AUC) was calculated. Normalized SmO_2_ values from the entire wall sit were plotted against time and SmO_2_ AUC was calculated via the trapezoidal rule.

## 3 Statistics analysis

**Time to Failure**: A paired-samples *t-*test was conducted to evaluate the effects of FoA on time to failure during the wall-sit tasks under both conditions (internal vs. external FoA).

**RPE**: A two-way analysis of variance (ANOVA) with two repeated measures (Condition: internal vs. external; Time: beginning vs. end) were conducted to determine differences in RPE between each condition. A Student-Newman-Keul's *post-hoc* test was used, if needed.

**Unpleasantness:** A two-way analysis of variance (ANOVA) with two repeated measures (Condition: internal vs. external; Time: beginning vs. end) were conducted to determine differences in unpleasantness scores between each condition. A Student-Newman-Keul's *post-hoc* test was used, if needed.

**Muscle Activation:** A two-way analysis of variance (ANOVA) with two repeated measures (Condition: internal vs. external; Time: beginning vs. end) were conducted to determine differences in muscle activation between each condition. A Student-Newman-Keul's *post-hoc* test was used, if needed.

**MDF:** MDF was assessed by a two-way analysis of variance (ANOVA) with two repeated measures (Condition, Time) were conducted to determine differences in EMG between each condition. A Student-Newman-Keul's *post-hoc* test was used, if needed.

**SmO**_**2**_**:** SmO_2_ (Δ BSL) was assessed by 2 × 2 mixed-factorial ANOVA with a within-subject factor of FoA condition (internal vs. external), and the phases of the trial (beginning vs. end). Additionally, the overall hypoxic stress was assessed by a paired *T*-test comparing the SmO_2_ AUC between conditions (internal vs. external).

## 4 Results

### 4.1 Effects of FoA on time to failure

**Figure 2:**
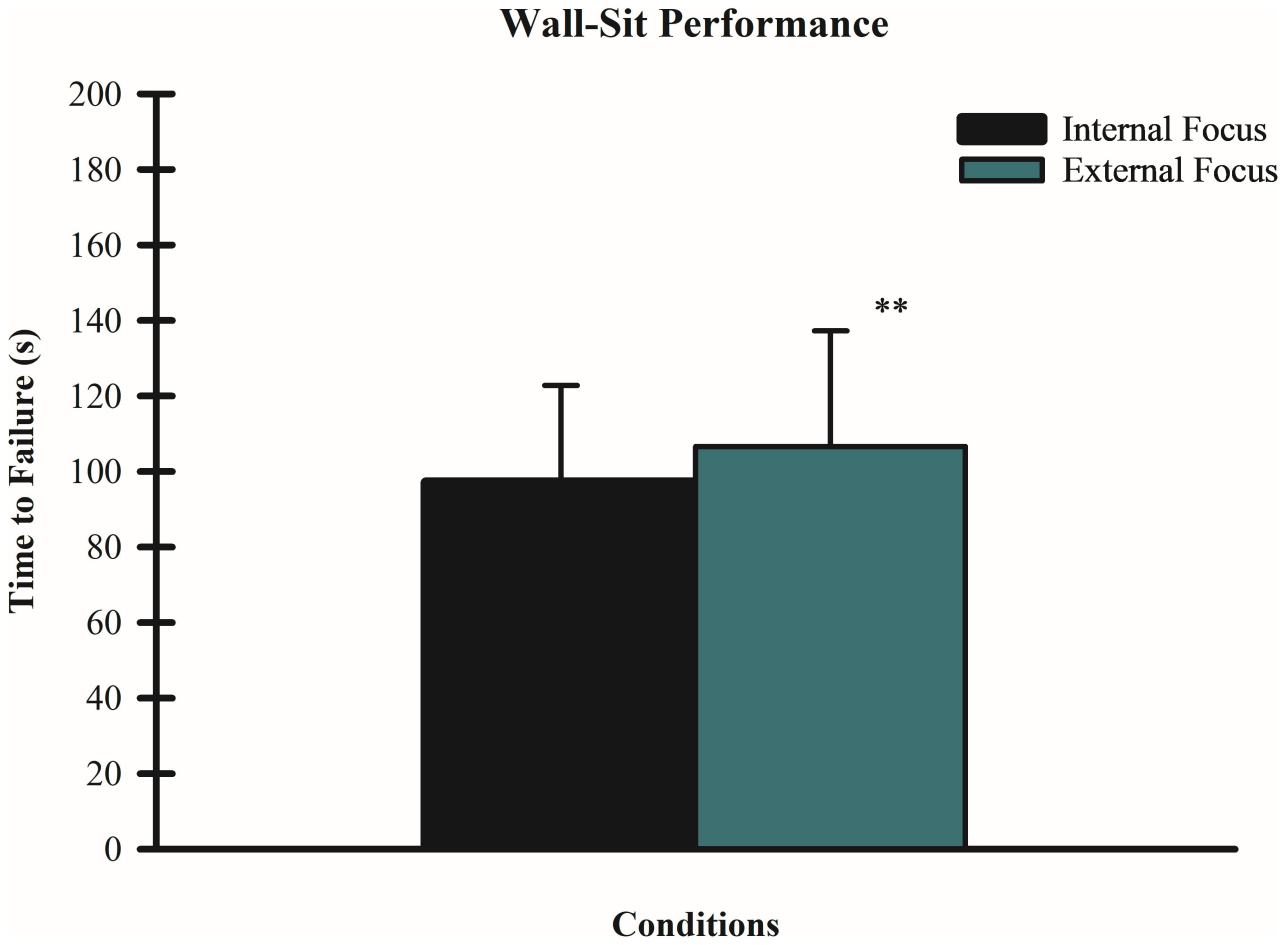
Effect of FoA on time to failure on wall-sit tasks. Mean time to failure in seconds during wall-sit tasks for both conditions (internal and external FoA) are shown. Error bars represent standard deviations. **p = 0.002 for the external FoA that allowed participants to stay longer in task relative to an internal FoA.

A paired-samples *t-*test was conducted to evaluate the effects of FoA on time to failure during the wall-sit tasks. Results indicated that there was a statistically significant difference between FoA conditions and time to failure *t*_(59)_ = −3.30, *p* = 0.002 (*d* = −0.27). Mean time to failure for the external FoA condition was 106.68 ± 30.50 s, while mean time to failure for the internal FoA condition was 97.06 ± 25.74 s. Therefore, it is possible to conclude that adopting an external FoA increased participants' time to failure during the wall-sit task relative to an internal FoA (see Figure 2).

### 4.2 Time to failure and participant characteristics

There was no statistically significant difference between the time to failure in each of the FoA conditions and the number of times that participants exercise per week *F*_(1, 59)_ = 0.109, *p* = 0.897, partial η^2^ = 0.103. This suggests that previous exercise frequency did not significantly impact wall-sit time to failure. Furthermore, no significant differences were found between time to failure in both FoA conditions based on whether subjects were student-athletes or regular students *F*_(1, 59)_ = 0.017, *p* = 0.897, partial η^2^ < 0.001. Therefore, being a student-athlete did not statistically impact wall-sit time to failure. Lastly, there was no interaction between time to failure, exercise frequency, or type of subject (student-athlete vs. regular student) *F*_(1, 59)_ = 0.127, *p* = 0.881, partial η^2^ = 0.072 (see Table 2).

**Table 2:** Time to failure and participant characteristics.

**Participant condition**	**Exercise frequency (days/week)**	**Subject type**	**Time to failure (mean)**	**Std. dev**.
Internal FoA	3–4 times	Student-athlete	108.43 s	14.35
		Regular student	83.59 s	24.99
		Total	87.73 s	24.96
	5–6 times	Student-athlete	112.86 s	24.90
		Regular student	101.35 s	26.32
		Total	102.36 s	26.04
	6 or more times	Student-athlete	106.55 s	19.27
		Regular student	88.26 s	24.45
		Total	97.06 s	25.74
	Total	Student-athlete	109.38 s	17.93
		Regular student	95.16 s	26.36
		Total	97.06 s^*^	25.74
External FoA	3–4 times	Student-athlete	115.56 s	23.96
		Regular student	87.13 s	22.40
		Total	91.87 s	24.19
	5–6 times	Student-athlete	117.30 s	23.05
		Regular student	113.05 s	33.28
		Total	113.43 s	32.25
	6 or more times	Student-athlete	117.42 s	28.13
		Regular student	99.03 s	27.71
		Total	102.97 s	27.82
	Total	Student-athlete	116.91 s	21.46
		Regular student	105.10 s	27.71
		Total	106.68 s^*^	30.59

A paired-sample t test revealed a statistically significant difference on time to failure during wall-sit conditions *t*_(59)_ = −3.30, *p* = 0.002 (d = −0.27) (Internal focus total: 97.06 s vs. External focus total: 106.68 seconds). Difference was represented by the “*.”

### 4.3 Effects of FoA on RPE during wall-sit tasks

**Figure 3:**
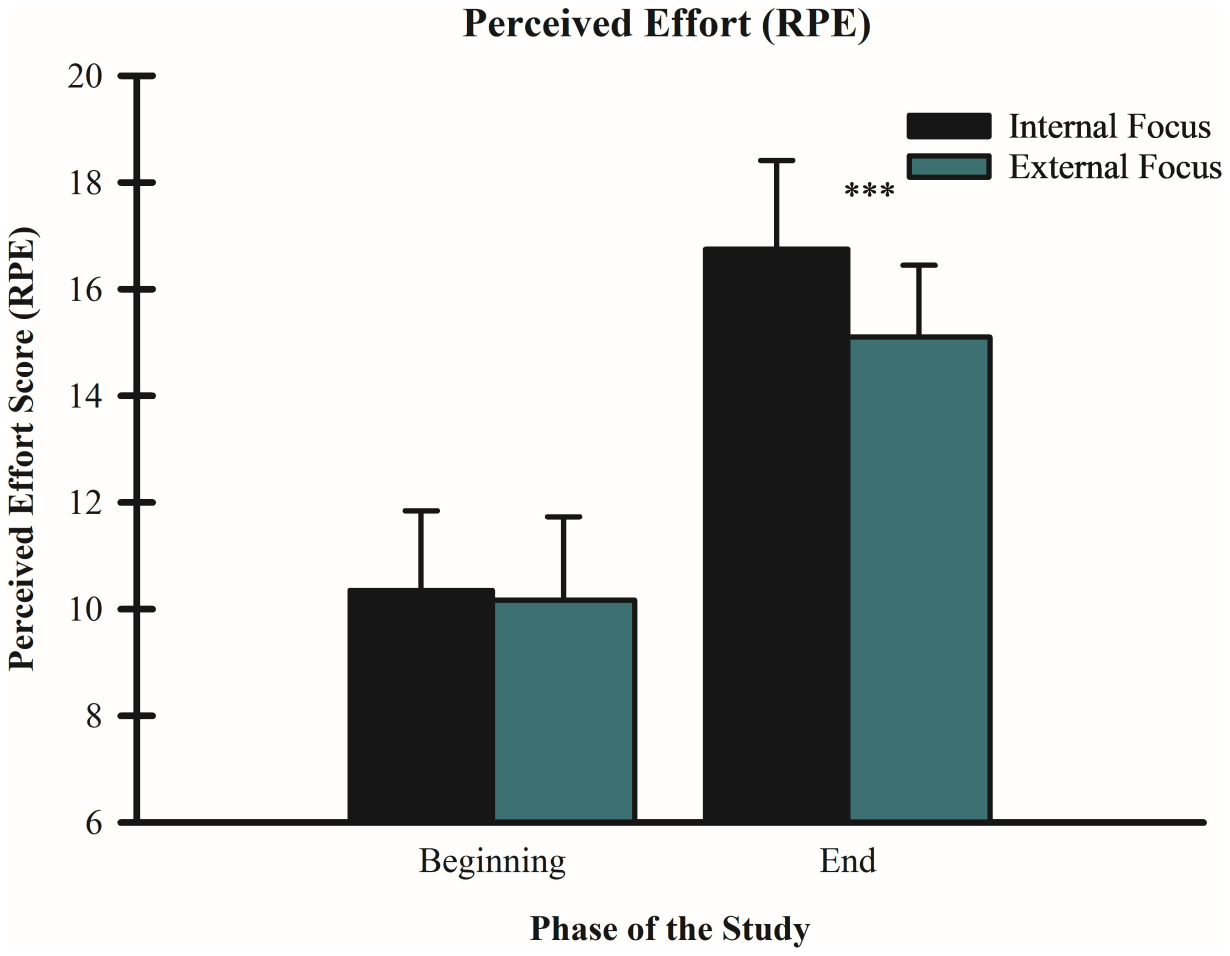
Effect of FoA on RPE. Mean RPE scores are shown for internal and external FoA conditions at the beginning and end of the wall-sit task. Error bars represent standard deviations. ***p < 0.001 for both internal and external FoA conditions (beginning vs. end).

A 2 (condition: internal vs. external FoA) × 2 (time: beginning vs. end) repeated-measures ANOVA indicated a significant main effect of time for both conditions, with RPE increasing from the beginning to the end of the wall-sit tasks: internal FoA, *F*_(1, 59)_ = 32.83, *p* < 0.001, partial η^2^ = 0.354 (beginning: M = 10.35, SD = 1.50; end: M = 16.75, SD = 1.66); external FoA, *F*_(1, 59)_ = 865.53, *p* < 0.001, partial η^2^ = 0.936 (beginning: M = 10.17, SD = 1.56; end: M = 15.10, SD = 1.35). The analysis also revealed a significant interaction between FoA condition and time, *F*_(1, 59)_ = 32.41, *p* < 0.001, partial η^2^ = 0.355. Importantly, as shown on the statistical analysis, RPE for the external FoA condition by the end of the wall-sit task was significantly lower than the internal FoA (see Figure 3).

### 4.4 Effects of FoA on affect (unpleasantness) during wall-sit tasks

**Figure 4:**
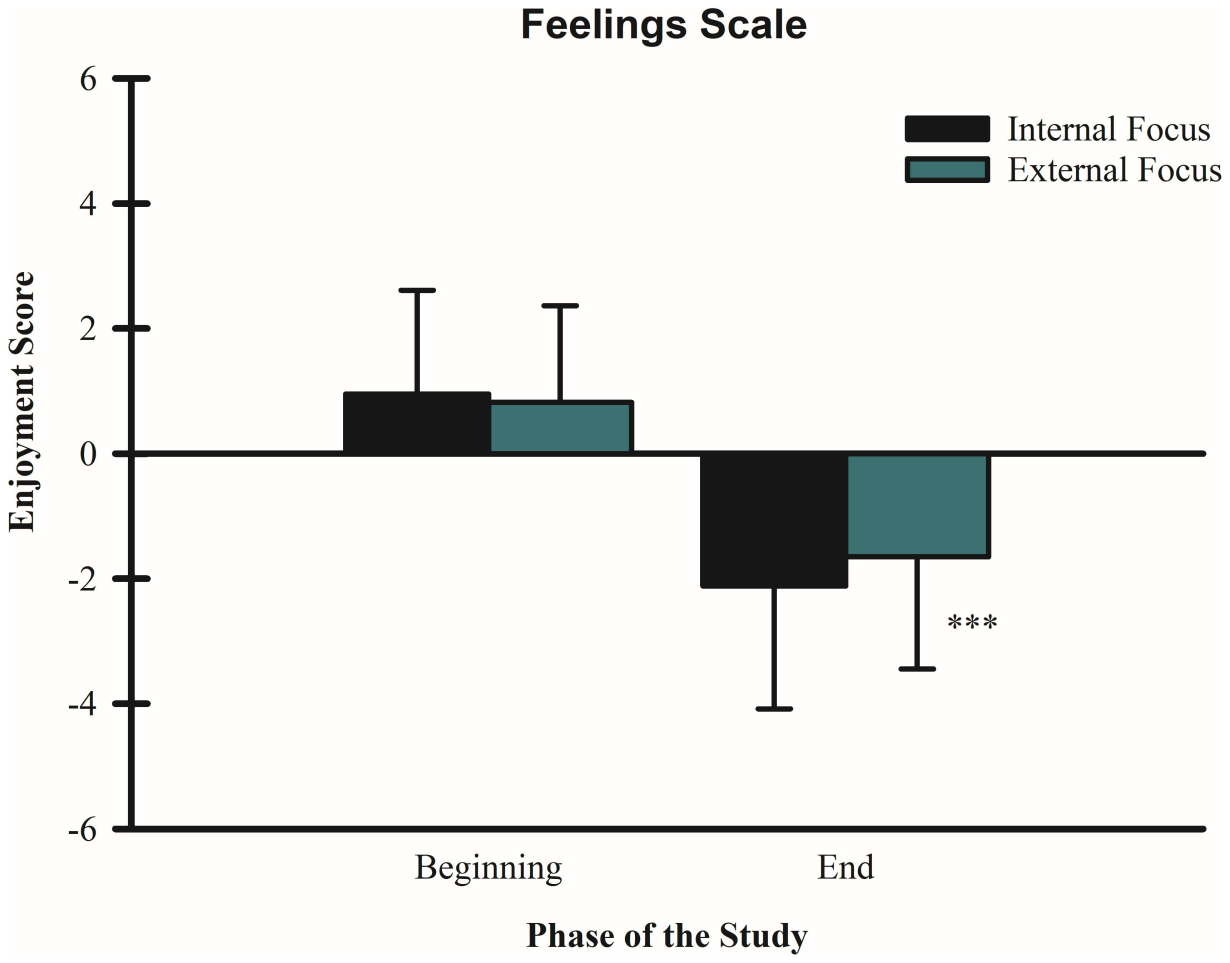
Effect of FoA on Unpleasantness. Mean pleasantness/unpleasantness scores are shown for internal and external FoA conditions at the beginning and end of the wall-sit tasks. Error bars represent standard deviations. ***p < 0.001 for the external FoA where individuals felt the wall-sit as less unpleasant than the internal FoA by the end of the trial. The scores ranged from +5 to−5, as +5 represents the highest pleasant feeling, and−5 the highest unpleasant feeling.

A 2 (condition: internal vs. external FoA) × 2 (time: beginning vs. end) repeated-measures ANOVA revealed a significant main effect of time for the external FoA condition, F_(1, 59)_ = 212.95, *p* < 0.001, partial η^2^ = 0.783 with unpleasantness increasing from the beginning (M = 0.82, SD = 1.54) to the end of the task (M = −1.65, SD = 1.79). No significant change across time was observed for the internal FoA condition, F_(1, 59)_ = 1.132, *p* = 0.292, partial η^2^ = 0.019 (beginning: M = 0.95, SD = 1.66; end: M = −2.12, SD = 1.96). The analysis further indicated a significant interaction between FoA condition and time, F_(1, 59)_ = 5.67, *p* = 0.020, partial η^2^ = 0.088. Specifically, it is possible to conclude based on the statistical analysis that when comparing both task performances (see Figure 4), by the end of the trials, individuals adopting an external FoA perceived the task to be less unpleasant than the internal FoA condition.

### 4.5 Effects of FoA on muscle activation during wall-sit tasks

**Figure 5:**
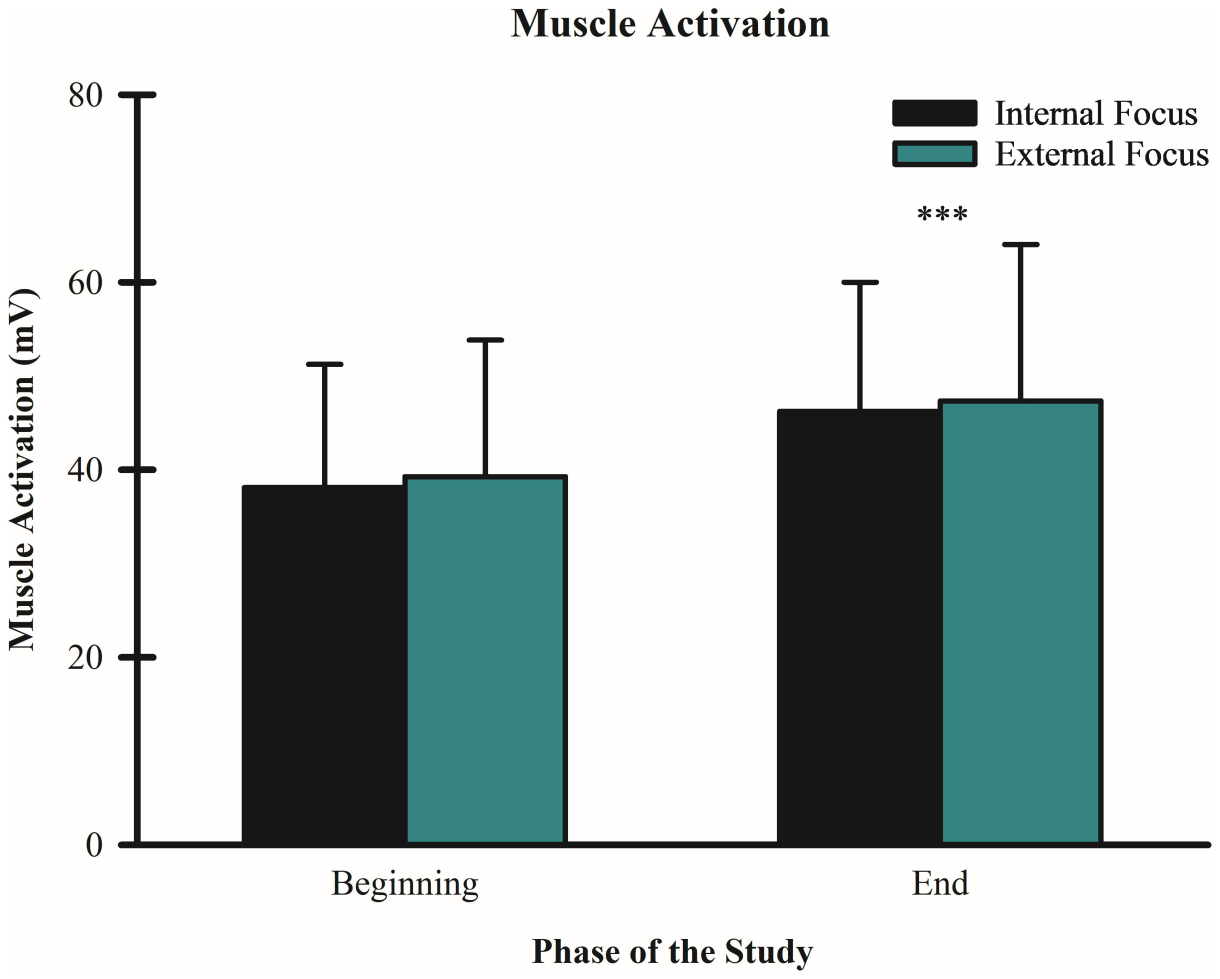
Effect of FoA on muscle activation during wall-sit tasks. Mean EMG amplitudes (V) are shown for internal and external FoA conditions at the beginning and end of the wall-sit tasks. Error bars represent standard deviations. There was a significant main effect for time, but no significant difference between conditions ***p < 0.001 for external FoA condition (beginning vs. end).

A 2 (condition: internal vs. external FoA) × 2 (time: beginning vs. end) repeated measures ANOVA was conducted to examine the effects of FoA on muscle activation (amplitude in volts) during the wall-sit tasks. Multivariate tests indicated a statistically significant difference in muscle activation with the external FoA condition across the beginning and end phases of the wall-sit tasks F_(1, 59)_ = 160.01, *p* < 0.001, partial η^2^ = 0.731. The mean amplitudes for the external FoA condition were 39.27 V (SD = 14.56) at the beginning and 47.35 V (SD = 16.70) at the end. By contrast, no significant difference was found for the internal FoA condition across the beginning and end phases F_(1, 59)_ = 2.42, *p* = 0.125, partial η^2^ = 0.039. Mean amplitudes were 38.11 V (SD = 13.14) at the beginning and 46.24 V (SD = 13.77) at the end. There was no significant interaction between FoA condition and phase of the wall-sit task F_(1, 59)_ = 0.002, *p* = 0.966, partial η^2^ < 0.001 (see Figure 5).

### 4.6 Effects of FoA on MDF

**Figure 6:**
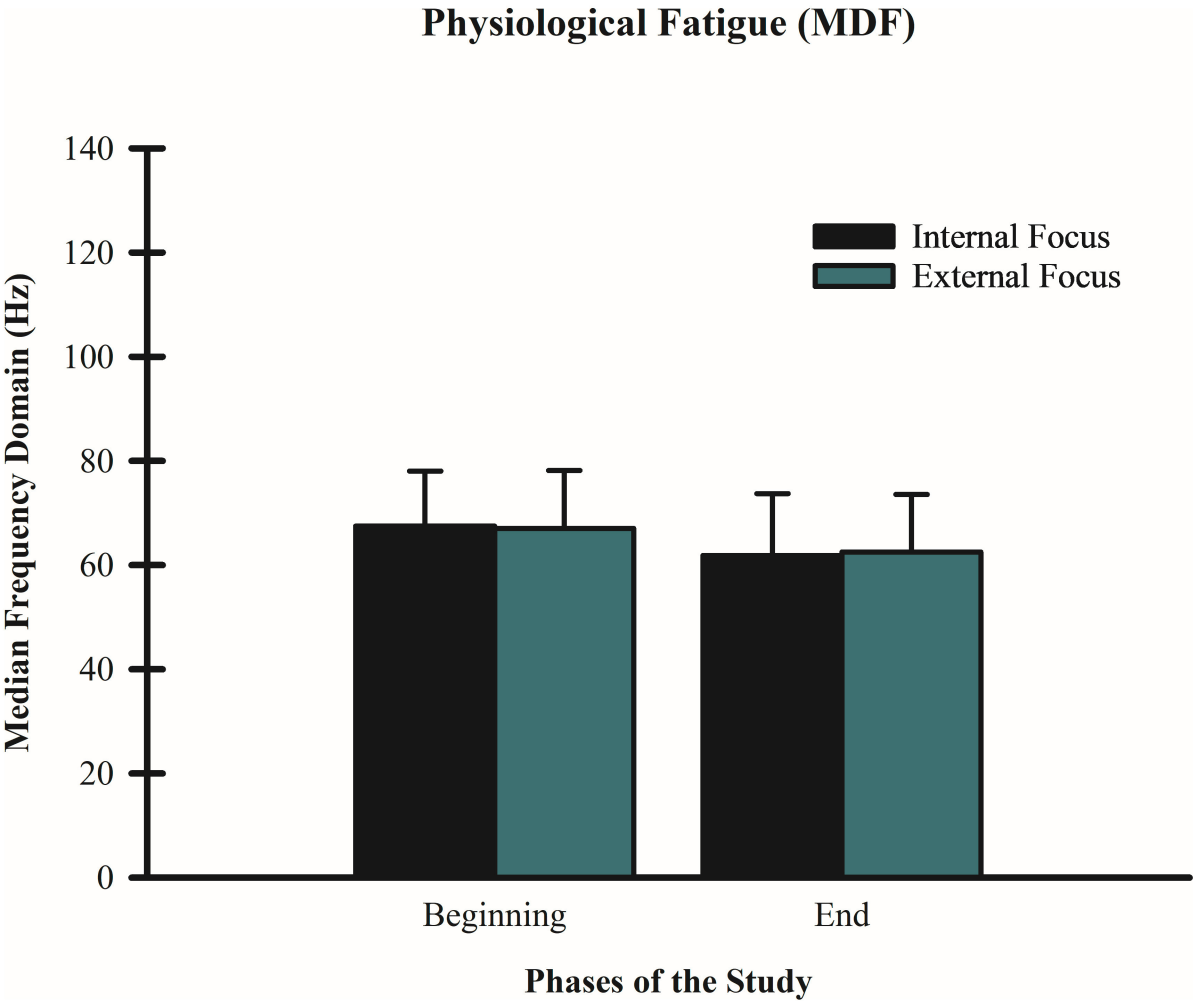
Effect of FoA on MDF. Mean MDF values (Hz) are shown for internal vs. external FoA over time. Measures of MDF for internal FoA was (M: 67.49, SD: 10.58) at the beginning and (M: 62.46, SD: 11.05) at the end. For external FoA, MDF was (M: 67.09, SD: 11.10) at the beginning and (M: 61.75, SD: 11.81) at the end. There were similar, significant increases in MDF over time for both FoA conditions (*p* < 0.001). Lower scores represent more fatigue.

A 2 (condition: internal vs. external FoA) × 2 (time: beginning vs. end) repeated-measures ANOVA for MDF revealed a significant main effect for time F_(1, 59)_ = 133.30, *p* < 0.001, partial η^2^ = 0.693, with MDF increasing from the beginning (M = 67.29, SD = 0.031) to the end of the wall-sit tasks (M = 62.10, SD = 0.031). However, there was no significant difference between FoA conditions F_(1, 59)_ = 1.26, *p* = 0.26, partial η^2^ = 0.021, and no condition by time interaction F_(1, 59)_ = 0.28, *p* = 0.59, partial η^2^ = 0.005 (see Figure 6).

### 4.7 Effects of FoA on SmO_2_

**Figure 7:**
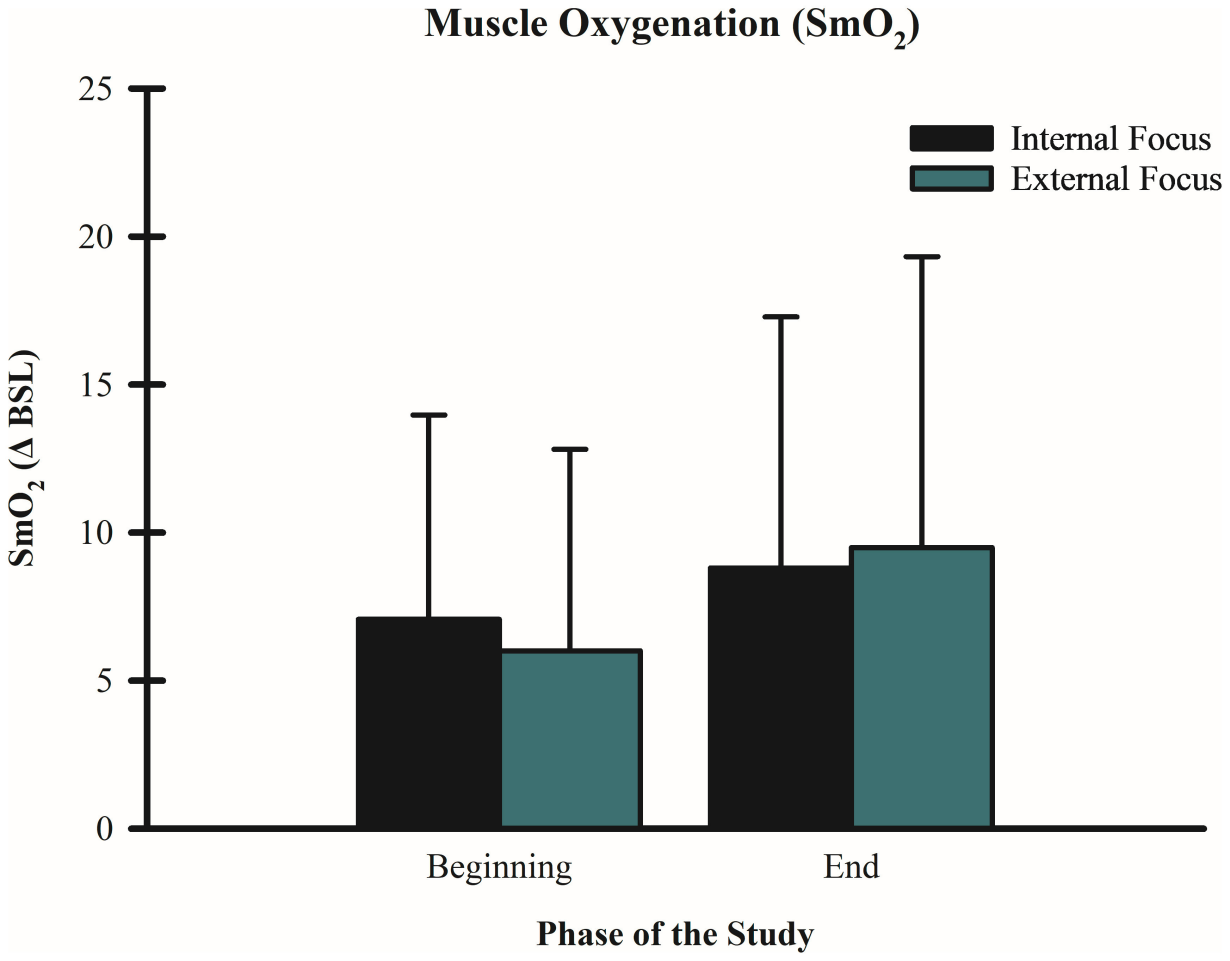
Effect of FoA on SmO_2_. Mean SmO_2_ values are shown for internal FoA vs. external FoA over time. For internal FoA, SmO_2_ was (M: 5.11, SD: 6.94) at the beginning and (M: 6.70, SD: 7.92) at the end. For external FoA, SmO_2_ was (M: 4.91, SD: 6.85) at the beginning and (M: 6.71, SD: 7.86) at the end. There was a similar, significant decrease in SmO_2_ over time (*p* < 0.001) in both FoA conditions.

A 2 (condition: internal vs. external FoA) × 2 (time: beginning vs. end) repeated-measures ANOVA for changes in SmO2 revealed a significant main effect for time F_(1, 59)_ = 17.10 *p* < 0.001, partial η^2^ = 0.284 with SmO2 decreasing from the beginning (M = 6.53, SD = 6.43) to the end of the wall-sit tasks (M = 9.14, SD = 8.89). However, there was no significant difference between FoA conditions F_(1, 59)_ = 0.005, *p* = 0.94, partial η^2^ = 0.001, and no FoA condition x time interaction F_(1, 59)_ = 0.077, *p* = 0.78, partial η^2^ = 0.023. A paired *T*-test for SmO2 AUC showed no significant difference *t*_(59)_ = 0.303, *p* = 0.763 (*d* = 0.171) between internal (M = 636.7, SD = 882.7) and external (M = 621.2, SD = 796.0) FoA conditions (see Figure 7).

## 5 Discussion

This study was inspired by Lohse and Sherwood (2011) experiment that investigated how FoA affected performance during an isometric and fatiguing task (wall-sit). The authors investigated time to failure and RPE. The current study tried to replicate (Lohse and Sherwood, 2011) and address potential physiological mechanisms underlying the superior performance of an external FoA on time to failure. We assessed MDF, muscle activation, and SmO_2_ as potential mechanisms to explain the effects of FoA on time to failure. Lastly, we added a measure of affect (unpleasantness) to understand individuals' perceptions while executing the wall-sit tasks. The utilization of physiological measurements such as EMG and NIRS was inspired by previous studies that showed how different FoA conditions disrupted or improved performance (Baumeister, 1984; Beilock and Carr, 2001; Lewis and Linder, 1997; Wan and Huon, 2005). These studies revealed that simply verbally prompting subjects to internally focus on their muscles, rather than focusing on the goal of the movement, or trajectory of the movement, increased errors, increased force production required to complete the task, and increased fatigue (Lohse et al., 2011).

In this experiment, we demonstrated that adopting an external FoA during an isometric and fatiguing task (wall-sit) prolonged participants' time to failure and their abilities to resist to fatigue. Additionally, during the external FoA condition, participants perceived the task as less fatiguing (lower RPE) and less unpleasant (lower score on the Feelings Scale) specifically when perceptions were assessed at the end of the wall-sit tasks, and close to failure. These results replicated the findings of Lohse and Sherwood (2011) that showed focusing externally increased subjects' time to failure. Importantly, in addition of assessing RPE, we examined unpleasantness during both conditions at the beginning and at the end of the wall-sit tasks. Our study revealed that the lower RPE in addition to the lower perception of unpleasantness during an external FoA, when assessed close to the time of failure, may have acted synergistically to attenuate the feelings and perceptions of fatigue that could be associated with the prolonged time to failure on the wall-sit tasks. This effect could be interpreted and associated to an enhanced ability to block or distract from the unpleasant sensations of MDF (muscle fatigue). Therefore, correlating the results of the muscle activation and MDF with the lower RPE and unpleasantness scores by the end of each condition, it can be suggested that an external FoA may elicit superior performance on a fatiguing task because it may act as a distractor/inhibitor of the negative effects of physiological alterations in participants' quadriceps. Interestingly, during the internal FoA condition, the subjects perceived and felt the negative effects of muscle fatigue more (high scores on RPE and Unpleasantness) making them fail to stay on task as long as the external condition, although both conditions resulted in similar MDF—physiological fatigue response.

It is important to mention that the benefits of external FoA on the wall-sit performance are not completely causal (“B is because of A”), but rather they may have associations with variables such as motivation, athletic performance, individual tolerance, and different aspirations. Additionally, it is crucial to highlight that perceived exertion, fatigue, and affect are not “all or nothing” variables. There are different processes, perceptions, and factors that impact the individual's interpretation and perception of the factors involved in performance. One of the most important theories related to fatigue and performance is the psychobiological model of endurance performance (Marcora, 2008). Specifically, the author suggests that exercise tolerance should be considered a conscious decision-making process based on an interaction between perception of effort, objective physiological alterations in fatigue, and potential individual motivation. The author suggests that fatigue is not only a physiological failure (e.g., lower scores, or higher muscle activation), but rather it is understood that while individuals are performing a fatiguing task, individuals may have different aspirations, motivations, and perceptions that positively and/or negatively impact the psychological response toward exercise, physical activity, and performance. In the same perspective between performance and fatigue, there is the “predictive processing theory” (Miller Tate, 2021). It suggests that fatigue is a top-down, protective process based on a conscious experience that is designed to prevent damage while performing. It balances expected effort with metabolic cost to generate performance. It takes into consideration individual perspectives/reasons when analyzing, perceiving, and feeling the variables related to the objective alterations of performance such as fatigue.

“Therefore, we understand the benefits of external focus of attention on performance (prolonged time to failure), decreased perceived effort and affect (unpleasantness), may be associated to some psychobiological factors involved in the task. For example, the conscious sensation of how hard, heavy, and strenuous a physical task is may be driven by individual differences. For example, perceived effort and affect may be associated with an individual's tolerance, past experiences, motivations etc. Additionally, on an individual difference perspective, we should assume that subjects may have different motives to participate in the study; it may be because they are student-athletes and want to ‘prove' they are great and may try harder, while others may participate in the study for the potential to earn extra points toward their course grade. Therefore, it can be assumed that individuals may try to persevere at differing levels at the first signs of negative consequences of the task, such as tremor in the lower limbs, pain, or burning sensations. Some participants may demonstrate a higher competitive personality trait and will try hard to sustain their position. All in all, we are proposing that physiological fatigue and its consequences may present differently and be impacted by differing personal psychobiological factors as presented by Marcora (2008) and Miller Tate (2021).”

Although only a few participants were familiar with the Focus of Attention and performance theory, this fact could also have impacted their willingness and motives to persist in the test. For instance, knowing that the external FoA enhances performance and learning, this could have impacted individuals' implicit motives to stay on task. This logic fits the self-fulfilling prophecy of the predictive processing theory that suggests that when you expect to feel tired you “feel more tired,” or when individuals expect to win the battle against fatigue they “won't feel tired.” In other words, knowledge that an external FoA may elicit superior results could have implicitly created more effort for some subjects to “justify the theory.” Additionally, participants that have a high tolerance for pain and are accustomed to performing fatiguing tasks may also have benefited during their performance in these tasks. For example, individuals who are athletes or have an athletic background may be able to persist on task a little longer. This factor falls within the proactive regulation of the predictive processing theory, because the brain uses prior experience to anticipate energy needs.

The results could also be interpreted following Ekkekakis (2003) findings. The author states that at higher levels of fatigue, bottom-up processes direct attention more strongly than top-down processes when performing skills, movements, and general motor tasks. For instance, as discomfort or fatigue increases, internal and bodily signals of pain will consume and dictate the attention resources, even when an athlete is trying to maintain an external FoA or focusing on something in the environment. Therefore, our experiment showed that adopting an external FoA may have overridden the bottom-up impulses and blocked the tendency of thinking internally when fatigue and discomfort was increased. The reduced RPE and unpleasantness affected individuals' performance by increasing time to failure during the strenuous task.

The current study also revealed a similar pattern of MDF during both FoA conditions, as both conditions saw signs of increased MDF toward the end of the wall-sit tasks (close to failure), but there was not a condition x time interaction. Therefore, different than previous findings (Lohse and Sherwood, 2012; Marchant, 2011; Marchant et al., 2009a,b; Lohse et al., 2010; Wulf et al., 2010) adopting an external FoA did not decrease MDF or muscle activation, but there was a longer time to failure. Finding an increase in MDF and muscle activation over time was expected because the longer individuals stayed on the wall-sit task, the more fatigued they would be expected to become. In addition, the longer individuals resist fatigue on an isometric task, a higher muscle activation is expected to help keep subjects on task. Different than in previous studies in the field of motor control, an external FoA in our study did not show a more efficient motor unit recruitment (lower muscle activation) as the subjects approached failure. Interestingly, Qin et al. (2025) investigated brain activity under different FoA conditions while subjects were requested to perform an arrow shooting routine. Results showed no statistical significance between FoA and motor performance. Additionally, compared to internal, external FoA showed greater neural efficiency. Furthermore, external focus enhanced activity in the visual cortex, while decreasing activity in the motor cortex. All in all, although there was not a statistically significant difference between focus of attention and performance of an arrow shooting routine, it was possible to see that the brain worked on a more efficient way—utilizing less cortical resources while maintaining similar performance. Additionally, Kuhn et al. (2021) focused on investigating the effects of foci of attention on motor pathways (slow vs. fast) while performing a pedal-tracking motor task. The authors found inhibition in the primary cortex when external focus was adopted, indicating a more spatial restriction of the motor command. Additionally, they found diminished activation in the slow(er) motor pathways that could explain why using an external focus is a more economic strategy for our brain. Both studies depicted a different result from our study. They showed a more efficient physiological behavior when performing motor skills under external focus, but did not show superior performance when this focus of attention was utilized. These results provide a potential interaction between the psychobiological factors that impact individual's performances. Focus of attention may not be the only determinant to impact performance and fatigue.

Lastly, we examined changes in SmO_2_ across FOA conditions and time (i.e., beginning vs. end of the wall-sit task). A significant main effect of time was observed, with greater deviations from baseline SmO_2_ values at the end of the wall-sit task compared to the beginning. SmO_2_ reflects the dynamic balance between oxygen delivery and utilization. The increased deviation from baseline at task completion is consistent with the physiological demands of the exercise: the anaerobic nature of the wall-sit task likely elevated oxygen consumption as fatigue developed, while the isometric contraction may have concurrently restricted oxygen delivery. Importantly, SmO_2_ serves as a proxy for metabolic stress, which is closely linked to muscular fatigue. Thus, the observed decline in SmO_2_ toward the end of the task aligns with expected fatigue-related metabolic changes. Despite differences in time to failure between FoA conditions, no significant differences in overall SmO_2_ were found between FoA conditions. This suggests that the external FoA condition may have elicited a slower rate of SmO_2_ decline, potentially delaying the onset of muscular fatigue. However, this interpretation remains speculative and warrants further investigation. Notably, this study is the first to explore the relationship between FoA and SmO_2_ during this type of wall-sit task. Future research is needed to elucidate how FoA influences SmO_2_ dynamics, and how these changes may impact fatigue, performance, and training adaptations associated with metabolic stress.

From an applied perspective, our study showed promising results for strength training, sports, and exercise training, because although there was no neuromuscular-related significant difference between conditions at the end of the wall-sit tasks, adopting an external FoA may have decreased perceived fatigue and unpleasantness leading to a potential longer time to failure. Within the coaching realm, most professionals provide an internal FoA as the primary attentional focus for performing different tasks, but these instructions appear to decrease performance (Durham et al., 2009; Porter et al., 2010). Therefore, there is strong evidence to suggest that an external FoA may be more beneficial across many skills and skill levels (Wulf, 2007b).

## 6 Conclusion

Results revealed that adopting an external FoA showed superior performance on the wall-sit task (prolonged time to failure) relative to an internal FoA. Additionally, external FoA reduced RPE and unpleasant affect during the task. No significant differences were observed for MDF, muscle activation, or SmO2 between FoA conditions. The superior performance for external FoA during the wall-sit task may be associated with reduced RPE and unpleasant affect. Thus, results suggest that external FoA may have led to improved performance during the fatiguing tasks due to psychobiological factors that may have reduced or alleviated the sensations of the physiological fatigue and muscle activation. These results have real-life applications for performers, coaches, and health professionals because instructions and feedback are normally provided in a way that emphasizes individual body movement corrections and to reinforce explicit monitoring actions (conscious focus). As noticed, internal focus resulted in inferior performance and increased negative perceptions of fatigue and affect. Therefore, professionals in physical therapy, rehabilitation clinics, and motor performance settings should refrain from encouraging the adoption of an internal FoA for their patients, clients, and athletes. Additionally, because energy gets depleted and metabolic alterations are expected during the practice of sports and motor performance, the adoption of an external FoA should facilitate and attenuate how performers perceive and deal with the implications of body reactions such as pain, burning sensations, and physiological fatigue amongst other factors. Results have replicability and applicability in different physical and athletic settings that may help performers improve their performance while decreasing sensitivity to the negative effects of fatiguing tasks.

## 7 Limitations and future research

Although one of the inclusion/exclusion criteria of this study was to have participants abstaining from exercising at least 12 h prior to data collection, we relied on their affirmative answer. This may have impacted fatigue and performance. Additionally, we required participants to perform both wall-sit tasks in the same day and gave them 10 min of rest between trials to avoid increasing fatigue on the second wall-sit task of the session. This interval may have impacted participants differently. In order to minimize this effect, we randomized the other of conditions to attenuate the potential effects of fatigue and to control for order of conditions. Another limitation of the study was that the antagonist (biceps femoris) activity was not recorded. Although co-contraction can contribute to joint stability, it is generally low in healthy young adults compared to older populations (Kubota et al., 2023), and was therefore not expected to substantially influence the outcomes of the present investigation. Nonetheless, the absence of antagonist EMG data prevents the assessment of any small differences in co-activation that may have occurred and should be considered for future studies. Furthermore, another limitation of this study was the fact that we did not utilize an electroencephalography (EEG) technique to investigate cognitive load (cortical resources allocation), to show whether participants in the internal focus of attention was focusing their attention on the task itself, by depicting higher frontal midline activation under prefrontal cortex, relative to the external focus, which is known to depicts lower activation under the same area due its automaticity and activation under motor cortex (Toolis et al., 2023; Cooke et al., 2014). Further, the current study aimed to investigate individuals' time to failure under two different FoA conditions in a single session experiment, whereas previous studies in the field of motor control analyzed learning and muscle efficiency during multiple days of activity. Our goal was not to study the effects of learning, but to understand the effects of FoA on physiological fatigue, time to failure, and physiological and psychological alterations, and mechanisms have been shown to be different for performance (1 day) and learning (multiple days). Future research should investigate the effects of focus of attention and performance with the utilization of an electroencephalogram (EEG) to analyze cortical resources allocation and brain activation for a more objective analysis of cognitive effort and brain activation. Specifically, we suggest that authors should analyze the EEG spectral power “Frontal Midline Theta” to understand cortical resources allocation on the prefrontal cortex that shows activation on working memory and anterior cingulate, that are known for showing a reliable measure for conscious processes and attentional capacity. Higher activation on Frontal Midline Theta suggests a key marker of internal focus of attention due to its focused attention, cognitive control, and mental effort representation. Therefore, higher activation on frontal midline theta may suggest that individuals are cognitively focused (explicitly monitoring) on the execution of the skill (Toolis et al., 2023; Cooke et al., 2014). Lastly, future should investigate the effect of FoA in different tasks such as dynamic strength training, or in sport applications such as soccer, volleyball, and tennis for example.

## Data Availability

The original contributions presented in the study are included in the article/supplementary material, further inquiries can be directed to the corresponding author.
